# Patient, Public, Consumer, and Community Engagement: From Consucrat to Representative Comment on "The Rise of the Consucrat"

**DOI:** 10.34172/ijhpm.2020.148

**Published:** 2020-08-15

**Authors:** Matthew DeCamp, Sarah E. Brewer, Vadim Dukhanin

**Affiliations:** ^1^Division of General Internal Medicine, University of Colorado, Aurora, CO, USA.; ^2^Center for Bioethics and Humanities, University of Colorado, Aurora, CO, USA.; ^3^Department of Family Medicine, University of Colorado, Aurora, CO, USA.; ^4^Department of Health Policy and Management, Johns Hopkins Bloomberg School of Public Health, Johns Hopkins University, Baltimore, MD, USA.

**Keywords:** Patient Engagement, Patient Participation, Representation, Healthcare Governance, Consumer

## Abstract

Patient, public, consumer, and community (P2C2) engagement in healthcare delivery, research, and policy-making has been long considered an ethical obligation and is increasingly a regulatory requirement globally. The requirement to include a P2C2 member on various governing bodies may have inadvertently created what Evelyne de Leeuw calls the "consucrat" – a career consumer who has been designated and professionalized to function on behalf of a particular group or community. The concept of a consucrat can be problematic when a P2C2 member is co-opted by an institution governing body or in situations where institutions only seek and listen to the same voice over time. In this commentary, we suggest that one way to avoid these problems is to take seriously the concept and process of representation. Representation is only meaningful when P2C2 members are actively connected with those whom they represent. Doing so helps ensure P2C2 members remain grounded in the real-world concerns of their constituency and that representatives, backed by the voices of others, will be more powerful in effecting change.

In a thoughtfully written perspective, Evelyne de Leeuw describes and critiques the “rise of the consucrat.”^1^ The consucrat can be understood as a career consumer identified by a healthcare institution and designated to function on its governing body in matters pertaining to health or health policy. Today’s phenomenon of the consucrat may be rooted in a decades-old belief that having consumers involved in healthcare planning and policy-making results in better outcomes. However, for de Leeuw, the ways in which consucrats have been designated and professionalized and by which they represent consumers at the interface “between street-level worry and institutional arrangements” is problematic. Only by fully recognizing the complex, multiple roles these individuals play and by supporting them can we expect consucrats to move up Arnstein’s ladder of participation to rise as partners in healthcare governance.^2^

Evelyne de Leeuw’s message effectively resonates with those who have been arguing for meaningful patient, public, consumer, and community (P2C2) engagement in health policy.^3-8^ In addition, de Leeuw joins others in offering measured critiques of the role of advocacy in theory and practice. Nearly two decades ago, Rebecca Dresser pointed out the promise and perils of advocacy groups’ involvement in biomedical research.^9^ In a more recent volume, Hoffman and colleagues offer a multidisciplinary analysis of the role of patients as policy actors.^10^ Like de Leeuw, they question whether patients or the public can achieve power or control atop Arnstein’s ladder, partly because of healthcare institutions’ unwillingness to cede power.

 In this commentary, we will first expand upon several key elements of de Leeuw’s analysis. Next, we suggest that the critique of designation, professionalization, and representation of consucrats is informative but incomplete. We argue that these three processes are not inherently problematic, even though they have been developed as imperfect solutions to the complex intersectional problems de Leeuw identifies. Lastly, drawing upon our research related to P2C2 engagement in healthcare governance in the US context, we will suggest a complementary solution to the problems evident from a consucrat discourse – one that expands engagement activities to include a broader-affected public.

## Consucrats vs. Advocates

For many, seeing P2C2 engagement in healthcare governance implemented in concrete legislation or requirements is a welcome sign of progress (and instantiates the fundamentals of healthcare as a human right^11^). Whether within healthcare organizations or at the broader policy level, such requirements are evident globally.^12-15^ Yet de Leeuw’s analysis reminds us of the unintended consequences that come with institutionalization of P2C2 engagement. Such requirements reflect the importance of engagement and its potential to impact policy. At the same time, however, compelling to designate a “consumer” member of governing body can inadvertently result in a situation where simply having the member present (or “checking the box”) suffices – a far cry from the ideal of engagement.

 This mentality might set up the dynamic that de Leeuw describes as the complex intersectionalities consucrats face. By being “the” patient, public, consumer, or community member of a governing body, there can be a tendency for the “consucrat” to inadvertently adopt an advocacy or even adversarial approach, whereby the “consucrat” must operate in dialectic fashion against the healthcare institution. As de Leeuw points out, this can be deeply problematic when the context is healthcare delivery or governance in general. Unlike disease-focused advocacy groups, who, for example, may very clearly and expectedly advocate for additional research funding or institutional resources (even if obtaining those detracts from others), the consucrat often has no obvious constituency and no singular focus.

 In practice, there can be substantive overlap in the goals, qualifications, and activities of “consucrats” and “advocates.” This means that conceptualizing “consucrats” as either perfectly analogous to, or perfectly distinct from, advocates or advocacy groups is problematic. “Consucrats” may sometimes function as types of advocates, but often do and should serve other roles.

## Designation, Professionalization, and Representation

 de Leeuw critiques three processes required for P2C2 engagement activities: designation, professionalization, and representation. More must be said regarding these processes in order to understand the difficult tradeoffs in each.

 By designation, de Leeuw means in part how P2C2 engagement participants are identified and chosen. The author gives an example of designation that is seen as a matter of benevolence, an act of kindness or good will on the part of healthcare institutions. Framing engagement as benevolent is indeed troubling in light of engagement’s roots in human rights and justice. The presented vignette – of a very well-connected and respected consumer who is familiar with the mental healthcare system and will “fill the consumer rep[representative] portfolio” – may seem almost farcical.

On one hand, we agree that such an individual is not an average citizen; on the other, there may be advantages to such a designation that should be considered. In the imperfect world of de Leeuw’s consucrat, who is troubled by power imbalances and who must employ pushback skills, choosing a well-connected and respected consumer may partly overcome intersectional challenges. Additionally, those who are also able to operate from within the system, such as physician advocates, may be able to bring both personal and professional values to bear in effective ways.^16^ In the formative stages of our research, for example, we found that retired healthcare executives who serve as patient members of healthcare organizations’ governance board could be quite effective at pushing and securing broadly positive, patient-centered changes in an institution (unpublished data). Moreover, when such an individual is connected with the broader patient population, as we suggest below, it may be possible to convey these voices even if that individual is far from a typical patient.

Because many recognize the obvious drawbacks that would come with designating only “powerhouse” types of individuals as consucrats, there has been a movement to create tangible ways to empower P2C2 engagement participants (along with increasing their number per each engagement activity).^17^ For example, orienting P2C2 members of governing bodies to their charge and how the body works, giving them background information (eg, about healthcare or research), and providing them with effective communication skills can all help P2C2 members be more effective. These efforts are laudable, and de Leeuw might place them under the category of professionalization, a process through which laypersons gain the knowledge and skills to become experts.

Professionalization, like designation, is neither intrinsically good or bad. To the extent that professionalization truly empowers laypersons to interact with and effect changes in healthcare, it can be good. To the extent that it results in co-optation of P2C2 voices, narrows the scope of what P2C2 participants can say, or results in situations where institutions only seek and listen to the same voice over time, it can be bad. However, we ought not to let fear of professionalization prevent us from taking efforts toward empowering P2C2 representatives. P2C2 engagement initiatives sometimes fall short in providing empowering representatives, leaving them ill-equipped to be effective representatives of the their communities or advisors to the institutions with which they are engaged.^18^ In these instances, failure to professionalize engaged laypersons amounts to tokenism and renders the promise of effective P2C2 engagement out of reach.

Finally, de Leeuw asks what (or whom) consucrats represent. This is arguably the central question of patient and public involvement in healthcare; in fact, how one designates and professionalizes consucrats should, in our opinion, be guided by this question. de Leeuw suggests that ideally the consucrat should be “heard speaking on behalf of, and representing, a group or community that shares particular value systems and is legitimately concerned about being heard and respected by some higher abstraction of organization.”^1^

However, this is only *one* answer to the question of representation. Our research examining what a general patient population expects of those who serve as members of patient and family advisory councils in the United States found representation to be of primary concern, but it also found no singular answer to what representation means.^19^ Some participants endorsed formal representation (where an individual is authorized to be a representative, eg, via election), others endorsed descriptive representation (where the representative is supposed to have characteristics in common with those represented), and still others were most concerned about substantive representation (where the representative is assessed by whether the interests of those represented are actually advanced).^20^

Both the participants and the literature stress the importance, as an extension of descriptive representation, of the lived-experience expertise of representatives. This not only adds legitimacy to representatives but also brings value to health systems; such experiential knowledge is complementary to the many other types of knowledge usually mobilized.^21^ Because each type of representation comes with its own practical implications for engagement activities, these findings suggest that much more work is needed to understand what representation does and should mean in different engagement contexts.

## An Alternative: From Consucrat to Representative

What can be done to improve P2C2 engagement in healthcare? de Leeuw suggests that “consucrat peak bodies” must provide greater support for consucrats. However, the centrality of representation leads us to believe this is not the only way. Rather than only focusing on how individual consucrats are designated and professionalized, there is an urgent need to broaden P2C2 engagement activities by taking concrete actions toward transforming consucrats to true representatives by connecting representatives to those whom they represent. In addition, we must identify and empower representatives from among communities and patient populations that we hope (and who deserve) to have represented. In our research, few patients knew they had representatives, and even fewer knew how to contact them; at the same time, nearly all thought they deserved to know and to interact with them.^22^ Similarly, in community-based work, grassroots representatives are ready to serve in formal roles of advising and partnering with healthcare systems and researchers but are rarely invited to engage with their local institutions.

 The expansion of engagement activities to connect representatives to those whom they represent can take different forms. Public meetings or “town hall” events could bring representatives into contact with their communities, or small group events could provide a more intimate setting for representatives to learn about needs, values, and priorities that should be brought forward. Representatives could send regular updates to their constituents, electronically or via post, and in turn request feedback from them that can inform how representatives work on behalf of their constituents.

Expanding P2C2 engagement in this way could help solve several of the problems de Leeuw identifies. This solution is grounded in the voice of P2C2 participants and is based on a patient-derived model of P2C2 engagement.^19^ When representatives are connected with those whom they represent, they can more easily convey more than their own individual views. Being connected to others can mitigate the adverse aspects of professionalization, such as cooptation, by ensuring representatives remain grounded in the real-world concerns of their constituency. Being accountable to one’s constituents can allow even a representative who is not an “average” citizen (but instead a “very well-connected and respected consumer”) to nevertheless speak on behalf of P2C2 broadly. Backed by the voices of others, the representative’s own voice will be more powerful in effecting change. This will lead P2C2 engagement practice out of the imperfect world of consucrats to a realm of representatives.

## The Role of Health Systems

 Of course, in order to realize this expanded model of P2C2 engagement, healthcare institutions must also change and adapt how they interact with and perceive these representatives. First, they must be more willing to cede power to their constituents and truly listen to the voices of P2C2 representatives. Without a willingness to hear and integrate those perspectives into the functioning of institutions, expanded representation will amount to more work with no additional tangible benefit in the health of people. Under this expended model, healthcare institutions recognize P2C2 representatives as partners in co-construction and co-creation and value their lived-experience expertise and the knowledge of context they can have. When this expertise is valued as equal and complementary to other knowledge and expertise, P2C2 representatives and institutions can work together toward improved healthcare.

Second, healthcare institutions have organizational ecological and ethical responsibilities to nurture cultural health capital among its professionals and bring institutional practices closer to the reality of those living with the diseases.^23,24^ This requires reaching to the most vulnerable in the populations they serve and bringing their perspectives through P2C2 engagement activities. It also requires revisiting and optimizing power balance in all forms of interactions with engagement participants.

Third, healthcare institutions must be willing to support the additional labor required to have effective representatives. This support may include financial compensation for these representatives and also effective orientation and on-boarding, as well as ongoing professionalization opportunities that allow them to act effectively within complex bureaucratic healthcare institutions and research enterprises. Importantly, implementing models for training and supervising representatives and evaluating engagement while avoiding tokenism requires an evidence-based approach.^25^ Many innovative examples are available to guide institutions in providing this infrastructure and support for P2C2 representatives.^26-30^

 We close by expressing our gratitude to Evelyne de Leeuw for stirring the field of P2C2 engagement research. We hope that this discussion will contribute to generating momentum that will ensure future successes of P2C2 engagement.

## Ethical issues

 Not applicable.

## Competing interests

 Authors declare that they have no competing interests.

## Authors’ contributions

 MD made substantial contributions to the conception of the work and drafted the initial manuscript. SEB and VD made substantial contributions to the interpretation of the concepts presented and revised the manuscript critically for important intellectual content. All authors approved the final version of the manuscript and agree to be accountable for all aspects of the work.

## Authors’ affiliations

^1^Division of General Internal Medicine, University of Colorado, Aurora, CO, USA. ^2^Center for Bioethics and Humanities, University of Colorado, Aurora, CO, USA. ^3^Department of Family Medicine, University of Colorado, Aurora, CO, USA. ^4^Department of Health Policy and Management, Johns Hopkins Bloomberg School of Public Health, Johns Hopkins University, Baltimore, MD, USA.
